# Anticancer potential of nanogold conjugated toxin GNP-NN-32 from *Naja naja* venom

**DOI:** 10.1590/1678-9199-JVATITD-2019-0047

**Published:** 2020-03-02

**Authors:** Saurabh S. Attarde, Sangeeta V. Pandit

**Affiliations:** 1 Evolutionary Venomics Laboratory, Centre for Ecological Sciences, Indian Institute of Science, Bangalore, Karnataka, India.; 2 Department of Zoology, Savitribai Phule Pune University, Pune, Maharashtra, India.

**Keywords:** GNP-NN-32, Anticancer, Toxin, Naja naja, Venom, Breast cancer, MCF-7, MDA-MB-231

## Abstract

**Background:**

Cancer is the second most common fatal disease in the world, behind cardiovascular disorders in the first place. It accounts for around 0.3 million deaths per year in India due to the lack of proper diagnostic facilities, prevention and treatment. Current therapeutic methods do not provide adequate protection and affect normal cells along with cancerous ones. Thus, there is a need for some alternative therapeutic strategy, preferably from natural products, which have been traditionally used for treatment of various diseases in the country.

**Methods:**

In this study, we have conjugated purified NN-32 toxin from *Naja naja* venom with gold nanoparticles and its anticancer potential was evaluated against human breast cancer cell lines. UV-Vis spectroscopy, dynamic light scattering, transmission electron microscopy, atomic force microscopy and zeta potential analysis were the techniques used for characterization of GNP-NN-32.

**Results:**

GNP-NN-32 showed dose- and time-dependent cytotoxicity against breast cancer cell lines (MCF-7 and MDA-MB-231). NN-32 and GNP-NN-32 induced apoptosis in both breast cancer cell lines. The results of CFSE cell proliferation study revealed that NN-32 and GNP-NN-32 arrested cell division in both MCF-7 and MDA-MB-231 cell lines resulting in inhibition of proliferation of these cancer cells.

**Conclusion:**

GNP-NN-32 showed an anticancer potential against human breast cancer cell lines. Analysis of detailed chemical characterization along with its cytotoxic property might help to perceive a new dimension of the anti-cancer potential of GNP-NN-32 that will enhance its biomedical function in near future.

## Background

Much innovative research has been carried out in recent years, and remarkable success has been achieved regarding the understanding of cancer development and its therapeutic treatment. Various therapies have been used for treating cancer including chemotherapy, radiotherapy, immunotherapy and gene therapy [1]. Currently, the use of chemotherapeutic drugs remains the predominant option for cancer treatment. One of the major drawbacks of using chemotherapeutics for cancer treatment is that some patients do not respond to the drugs and eventually develop resistance with high toxicity. Furthermore, anticancer therapy is generally costly [1]. This has led to a search for alternative anticancer agents, ideally from natural resources, such as snake venom.

The use of cobra venom for treatment of cancer in mice was examined by Calmette et al. in 1933 [2]. The potential of natural products - such as venoms and their purified compounds - to fight cancer has drawn interest from all over the world. Indian spectacled cobra (*Naja naja*) venom increased the lifespan of Ehrlich ascites carcinoma (EAC) bearing mice, showing cytotoxicity on EAC cells and normalizing the antioxidant profile. The venom also showed cytotoxic activity towards human leukemic cell line U937 through the apoptotic pathway [3]. *Hydrophis spiralis* snake venom showed antitumor activity on EAC bearing mice by increasing the lifespan of treated mice as well as decreased tumor growth. *H. spiralis* venom caused DNA fragmentation and increased caspase 3 production, which is the central executioner of apoptosis [4]. NN-32 purified from *Naja naja* possessed cytotoxic activity against MCF-7 and MDA-MB-231 cell lines [5] and against EAC in BALB/c mice [6].

Recent finding on venom activity reported that snake venom toxins showed promising results against various experimental pathophysiological conditions such as leukemia [7], arthritis [8], stroke [9], neural trauma, Alzheimer’s disease, Parkinson’s disease [10] etc. Despite promising results against different pathophysiological conditions, the toxins failed to cross the barrier of safety line due to their toxicity. That is one of the major drawbacks for their progress in the drug development process. Therefore, research is carried out to overcome this limitation of the potential toxins. Several attempts including liposome encapsulation and silica coating had been made [11]. Recently, several toxins have been conjugated with nanoparticles to increase their functional efficacy. It is also reported that nano-conjugation reduces the toxicity of bioactive molecules by limiting their non-specific binding [12, 13, 14], which constitutes a promise to overcome the toxicity drawback of bioactive toxin molecules.

Nanotechnology is a dynamic domain for drug development in biomedical science. It is expected to play a vital role not only in molecular imaging, as biomarkers and biosensors, but also in drug delivery and gene therapy [15]. Thus, we have used a method called nano-conjugation that involves conjugation of the NN-32 toxin with gold nanoparticles. Nanoparticles (NP) can deliver a drug to the desired target; this ability of NP is responsible for its therapeutic and diagnostic use. Thus, by conjugating NP with toxin we are making them recognizable as the biological targets. Nano-conjugation is preferably done by using gold because it does not show any sensitivity towards light and air and it is unreactive to other molecules. However, gold tends to form bonds with itself and, thus, in order to keep them apart; their surfaces must be covered with a layer of polyethylene glycol (PEG) as protective molecules. Capping GNP with PEG enhances their stability and biocompatibility [16].

Studies have revealed that gold nanoparticles show particle distribution dependent upon the size. Hence, small nanoparticles show maximum dispersion throughout organs. Spermatotoxicity of gold nanoparticles has been detected and reported [17]. Aggregation of gold nanoparticles in red blood cells (RBC) with no significant RBC damage has been reported raising questions about possible implications of nanotoxicity during chronic exposure [18]. Reports of gold nanoparticle-induced oxidative damage in lung fibroblast cells *in vitro* are documented [19]. Pharmacokinetics of gold nanoparticles *in vivo* is also essential to assess their absorption, biodistribution, metabolism and elimination processes. Zhang et al*.* [20] reported that of the three administration routes for gold nanoparticles within the animal system, the oral and intraperitoneal ones showed higher toxicity and intravenous showed lower toxicity. Therefore, the present study aims at exploring the conjugation of NN-32 with gold nanoparticles, as well as characterization and anticancer potential of GNP-NN-32.

## Methods

## Chemicals

Dulbecco’s modified eagle medium (DMEM), fetal bovine serum (FBS), penicillin, streptomycin and trypsin-EDTA solutions were bought from Himedia (India). 3-(4,5-dimethylthiazol-2-Yl)-2,5-diphenyltetrazolium bromide (MTT), dimethyl sulphoxide (DMSO), neutral red (NR), formaldehyde, β-nicotinamide adenine dinucleotide reduced dipotassium salt (β-NADH), trypan blue, triton X-100 dye solution, gold chloride, polyethylene glycol(PEG) and sodium borohydrate were purchased from Sigma-Aldrich (United States). Dead cell apoptosis kit with Annexin V FITC/ PI and CellTrace™ CFSE Cell Proliferation kit were purchased from Thermo Fisher Scientific (United States).

## Preparation of GNP-NN-32

A protein toxin NN-32 was purified from *Naja naja* venom using ion exchange chromatography and RP-HPLC [5]. Sodium borohydride reduction method [21]was used with some modifications to synthesize gold nanoparticles. 20 mM HauCl_4_ (200 μL) and 10 mg/mL PEG (10 μL) were mixed with 10 mM of sterile phosphate buffer (800 μL, pH 7.2). Then 100 mM NaBH_4_ (40 μL) was added dropwise and stirred at 37°C for 1 h. The color of the reaction mixture was yellow and after several minutes, it changed to ruby red after mixing the NaBH_4_. In the properly controlled conditions, a synthetic method was developed to produce stable gold nanoparticles. For preparation of GNP-NN-32, 832 μL purified protein toxin NN-32 (1 mg/mL) was added in the systemized gold nanoparticle solution and kept for proper conjugation at 37°C for 24 hours [16].

## Characterization of GNP-NN-32

### *UV-VIS spectroscopy*

UV-VIS spectroscopy (Jasco V-630, Japan) measurements from 200 to 700 nm and 400 to 700 nm were performed for GNPs, NN-32 and GNP-NN-32. Spectra were taken with a special resolution of 1 nm at room temperature using 1 cm optical length cuvette [22].

### *Dynamic light scattering study*

Twenty-four hours after the preparation of conjugation, the cuvette was placed in the dynamic light scattering (DLS) apparatus. DLS measurements were performed in a Sympatee apparatus (NANOPHOX NX0088, Germany) at 25°C [22].

### *Transmission electron microscopy study*

The morphology and size of nanoparticles were examined by transmission electron microscopy (TEM). The GNP and GNP-NN-32 solutions were dropped on standard carbon-coated copper grids (200-mesh) and air dried for about 2 hours. The TEM images were obtained using a JEOL JSM 1100 transmission electron microscope, operating at 80 kV. Size distribution of the nanoparticles was determined based on TEM images.

### *Atomic force microscopy (AFM) analysis*

A drop of the sample solution was placed on a glass slide and allowed to dry for 2 hours. AFM images were obtained using the atomic force microscope (JEOL JSPM-5200, Japan) in a dark room at 25°C. The cantilever oscillation frequency was tuned to the resonance frequency of approximately 256 kHz [23]. 

## Zeta Potential

The zeta potential analysis was done using Backman Coulter Delsa Nano Zetasizer, USA. The pH of solution was adjusted to 7.2. The Smoluchowski approximation was used for the calculating zeta potential, because an aqueous solution was used to measure electrophoretic mobility [24]. 

## Estimation of NN-32 per GNP

UV-Vis titration was performed for calculating the number of peptides per GNP (1 mM) using a range of NN-32 concentration from 10 μg/mL to 100 μg/mL. The NN-32 and GNP solutions were incubated at room temperature for 30 min. The GNP was removed by 0.2 μm filter (Himedia) and the concentration of remaining free peptides in the solution was measured from the absorbance at 280 nm. From a linear fit of data points, the number of NN-32 per GNP was calculated [25]. 

## ***In vitro* Release Kinetics**

One milligram of GNP-NN-32 was added in 1 mL of PBS (pH 7.2) and incubated with light agitation of 150 *g* in an incubator shaker at 37^o^C for 7 days. After every 24 hours, individual samples were collected by centrifugation at 10,000 *g* for 20 min. The NN-32 release in the medium was determined by UV-Vis spectrophotometer at 280 nm. All measurements were performed in triplicates [26]. 

## Cytotoxicity Study

### *Cell culture*

Human breast cancer (MCF-7 and MDA-MB- 231) and human normal breast epithelial (MCF-10A) cell lines were acquired from the National Centre for Cell Sciences (NCCS), Pune, India. For culturing of these cell lines, DMEM supplemented with 10% heat inactivated FBS, penicillin (100 units/mL) and streptomycin (10 mg/mL) were used. Cells were grown in a CO_2_ incubator to sub-confluence at 37°C with 5% CO_2_.

### *MTT assay*

MCF-7, MDA-MB-231 and MCF-10A cells in the density of 1 × 10^4^/well were seeded into 96-well plates and incubated at 37°C under 5% CO_2_ for 24 hours. The cultured cells were then treated with doxorubicin (0.5-5 μM) as the positive control group and GNP-NN-32 (0.125-16 μg/mL) for 48 hours. After incubation, 20 μL of MTT solution (5 mg/mL) was added to each well and further incubated for 4 hours. The formazan precipitate formed by live cells was dissolved in DMSO. The absorbance of the mixtures was determined using microtiter plate reader at 570 nm and the cell viability was expressed as percentage inhibition relative to controls. All experiments were performed in triplicates. The dose-response curve was generated for each cell line to determine IC_50_ value [5]. 

## Anti-proliferation Assay

Anti-proliferation assay on MCF-7 and MDA-MB-231 cells were performed using the protocol mentioned in Attarde and Pandit [5]. Both cells were seeded at density of 1 × 10^4^ cells/well into 6-well plates and incubated for 24 hours. Then, these cells were treated with 5, 10 and 15 μg/mL of GNP-NN-32 for 24, 48, and 72 hours at 37°C under 5% CO_2_.

## Neutral Red Uptake Assay

The neutral red uptake assay was performed using the protocol reported in Abdullah et al. [27]. The cells were treated with different concentrations of GNP-NN-32 and plates were incubated for 24, 48 and 72 h under 5% CO_2_ at 37°C and then washed three times with 200 μL of PBS. Plates were further incubated in medium containing 200 μL NR solution at 25°C for 3 h, and then the cells were subsequently washed to remove the NR solution. Cells were then exposed to fixing solution consisting of 1% CaCl_2_and 0.5% formaldehyde in deionized water for 2 min followed by two washes with 1% acetic acid and 50% ethanol in deionized water. The plates were incubated for 10 min and then read in a microplate reader at 540 nm.

## Apoptosis Study by Flow Cytometry

Flow cytometric analysis using Annexin V FITC and propidium iodide (PI) was done to distinguish among live, apoptotic and necrotic cells after treatment of MCF-7 and MDA-MB-231 cells with NN-32 (IC_50_) and GNP-NN-32 (IC_50_). About 10^6^ cultured cells (MCF-7 and MDA-MB-231) were treated with NN-32, GNP-NN-32 and doxorubicin as a standard drug for 48 h. The cells were then washed with cold PBS and centrifuged at 1500g for 5 min at 4°C. The cells were resuspended in 1X Annexin-HEPES buffer and washed twice. The pellets were resuspended in the same buffer (100 μL). Annexin V-FITC (5 μg) and propidium iodide (1 μg) were added to the cell suspension. After 15 min of incubation in dark at room temperature, the analysis was done by flow cytometry (Thermo Fisher Attune NxT, USA). Flow cytometric reading was taken using 488 nm excitation and bandpass filters of 530/30 nm (for FITC detection) and 585/42 nm (for PI detection). Cells that were PI negative and Annexin V negative were considered alive cells, PI negative and Annexin V positive cells were considered apoptotic, and cells that were positive to PI and Annexin V negative were considered necrotic.

## CFSE Cell Proliferation Study

The cell proliferation study was performed using CellTrace™ CFSE Cell Proliferation Kit from Thermo Fisher scientific (USA). CFSE is a fluorescent dye that is used for flow cytometric monitoring of cell division. The non-florescent dye passively diffuses across the cell membrane and is cleaved by intracellular esterases within the viable cell. The cleaved dye becomes highly fluorescent and covalently binds to protein amine group within the cell. As the viable cell divides, CFSE dye is distributed uniformly among daughter cells, each daughter cell acquires approximately half of the CFSE intensity of its parent cell.

Cultured MCF-7 and MDA-MB-231 cells in 10^6^/mL were divided into different groups such as control, doxorubicin treated, NN-32 treated and GNP-NN-32 treated. One microliter of CellTrace™ stock solution in DMSO was added to each mL of cell suspension in PBS for a final working solution. Cells were incubated for 20 minutes at room temperature or 37°C, protected from light. Five times the original staining volume of culture medium (containing at least 1% protein) was added to the cells and incubated for 5 min. This step is for removal of any free dye remaining in the solution. Cells were pelleted by centrifugation and resuspended in fresh pre-warmed complete culture medium. Then, they were incubated for at least 10 min before analysis to allow the CellTrace™ reagent to undergo acetate hydrolysis. The next step consisted of cell stimulation, incubation, or analysis. Cells were incubated for a period of 5 days at 37^o^C with 5% CO_2_. They were analyzed using a flow cytometer with 488 nm excitation and emission filters appropriate for fluorescein at day 1 and after 5 days of incubation.

## Statistical Analysis

For the number of experiments conducted, data are depicted as mean ± SD. To evaluate the difference between two independent groups of samples paired Student’s t-test was performed. The repeated measure analysis of variance (ANOVA) was used to determine the significant differences between groups. In all analyses, *p* < 0.05 was considered statistically significant.

## Results

## Preparation of GNP-NN-32

The gold nanoparticles were synthesized and NN-32 (1 mg/mL) was added into the reaction mixture, resulting in a light purple colored colloidal solution. The solution was then kept stable at room temperature (25±2°C) and pH 7.2 for 40 days.

## Characterization of GNP-NN-32

### *UV-VIS spectroscopy*

**Figure 1:**
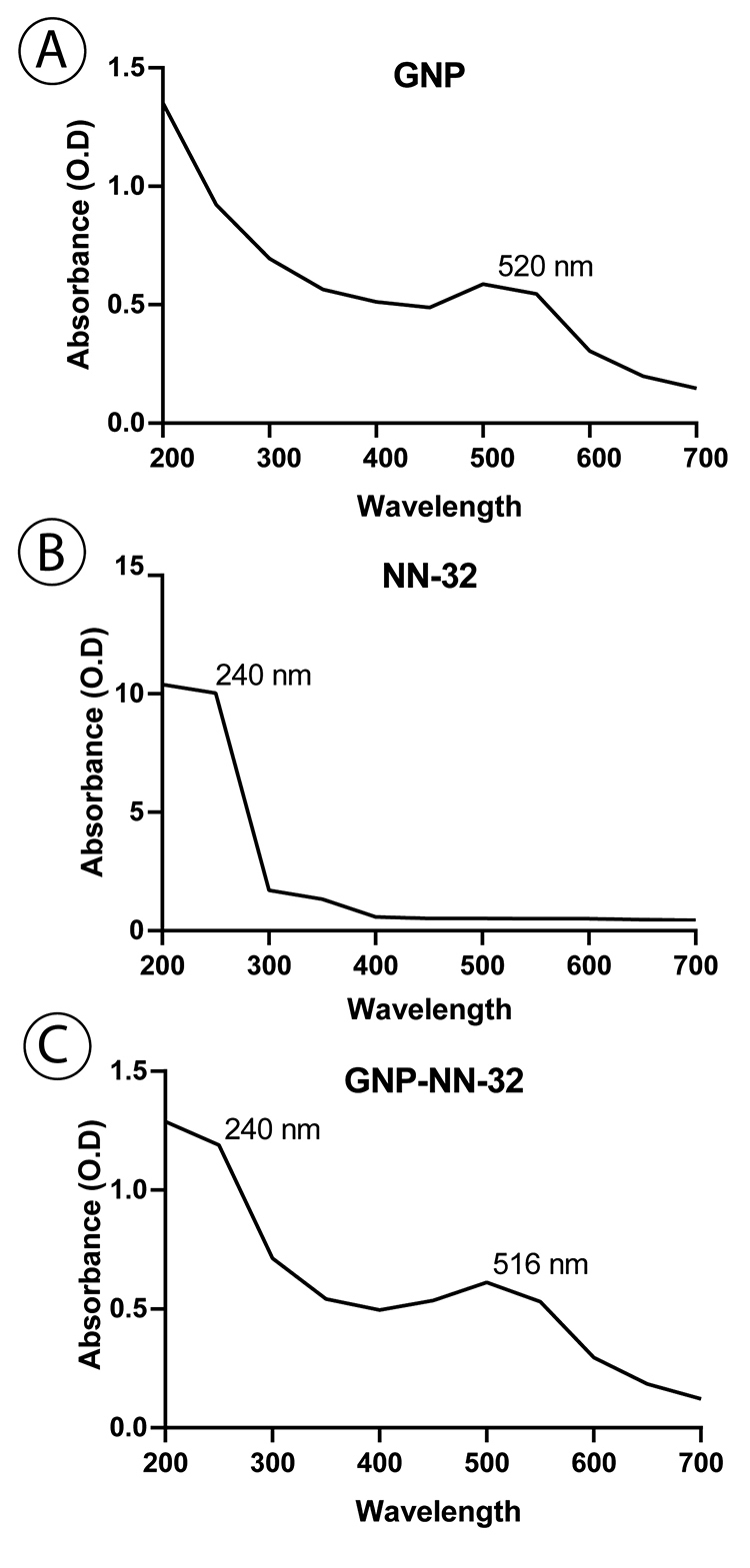
Characterization of GNP-NN-32 by UV-VIS Spectroscopy. **(A)** Absorbance spectra of GNP (λmax = 520 nm). **(B)** Absorbance spectra of NN-32 (λmax = 240 nm). **(C)** Absorbance spectra of GNP-NN-32 (λmax = 516 nm).

The plasmon resonance of NN-32 was formed at the 240 nm, having the characteristics of peptide molecule. Bare gold nanoparticles showed a peak at 520 nm and NN-32 showed a peak at 240 nm. The Plasmon resonance of GNP-NN-32 was formed at 516 nm, which confirmed the conjugation (Fig. 1). 

### *Dynamic light scattering study*

**Figure 2:**
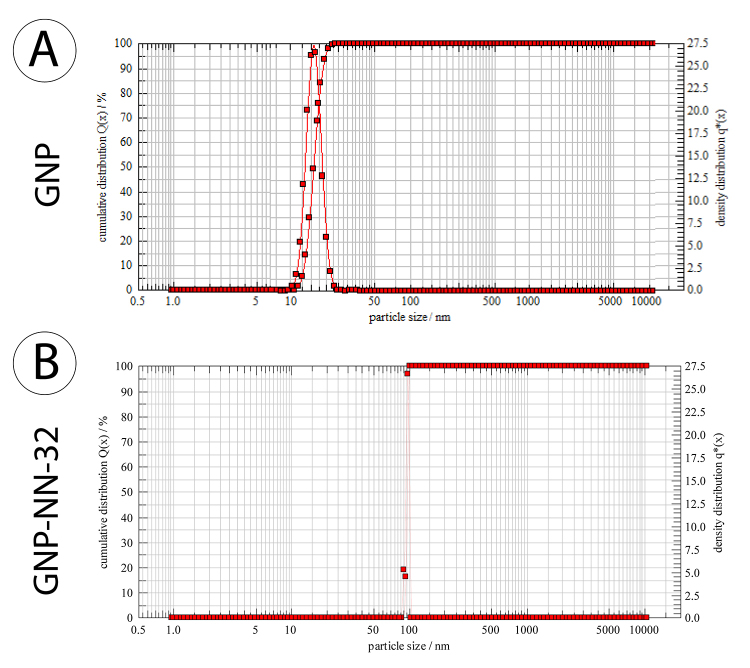
Characterization of GNP-NN-32 by DLS. **(A)** GNP, **(B)** GNP-NN-32.

The hydrodynamic diameter of the GNP measurement with DLS was observed to be 10-25 nm, with an average size of 18 nm. The hydrodynamic diameter of GNP-NN-32 was found to be 90-99 nm, with an average size of 95 nm (Fig. 2).

### *Transmission electron microscopy study*

**Figure 3:**
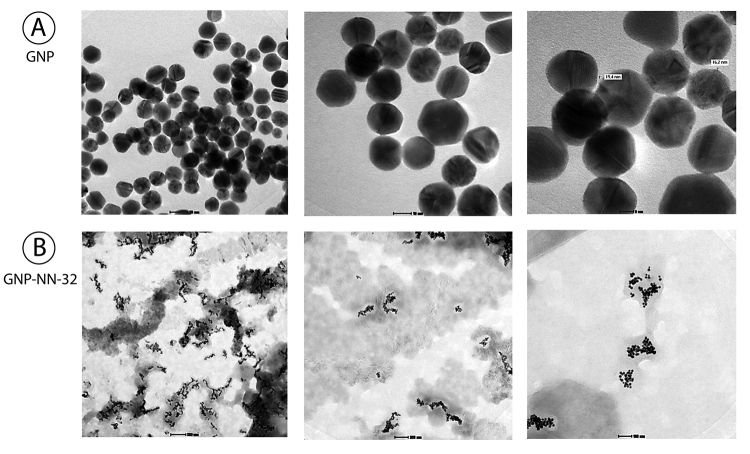
Characterization of GNP-NN-32 by TEM. **(A)** GNP, **(B)** GNP-NN-32.

A drop of the sample was placed on the copper grid and allowed to dry overnight. As observed a protein covered the whole surface of the nanoparticles and increased the hydrodynamic size of the NN-32 capped gold nanoparticles (Fig. 3).

## Atomic Force Microscopy (AFM) Analysis

**Figure 4:**
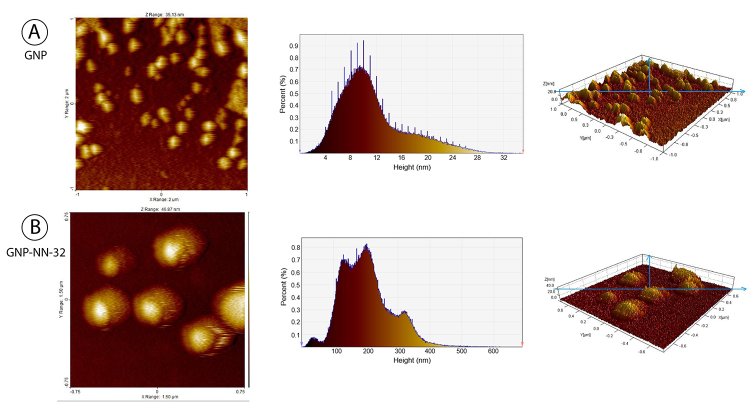
Characterization of GNP-NN-32 by AFM. **(A)** GNP, **(B)** GNP-NN-32.

GNP diameter determined by AFM was found to be 2-30 nm and GNP-NN-32 diameter was found to be 50-250 nm (Fig. 4)

## Zeta Potential

The measured zeta potentials of GNP, PEG capped GNP and GNP-NN-32 at pH 7.0 were -19.80, -18.90 and -12.6 mV, respectively. Stability of GNP was increased to some extent after PEGylation and conjugation with NN-32.

## Estimation of NN-32 per GNP

**Figure 5:**
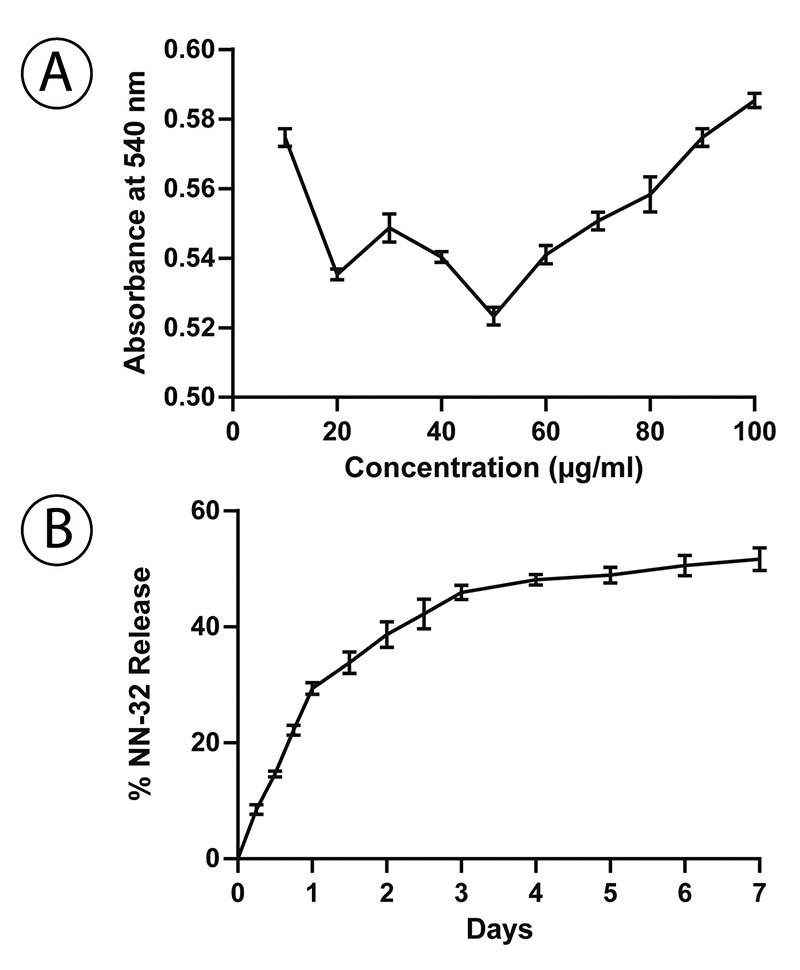
(A) Estimation of NN-32 per GNP. (B) *In vitro* NN-32 release profiles from GNP. GNP-NN-32 showed biphasic release kinetics.

The number of peptide per nanoparticles was calculated by UV-Vis spectroscopy of GNP using increasing concentration of NN-32 (10 µg/mL-100 µg/mL). Lowest absorbance at 540nm confirmed that 50 µg/mL NN-32 was the maximum concentration of 1 mM of gold nanoparticles (Fig. 5A).

## ***In vitro* Release Kinetics**

This study was carried out using UV-Vis spectroscopy. GNP-NN-32 showed biphasic release kinetics, a faster release profile wherein about 30% of the drug was released in 24 hours and thereafter 50% of NN-32 was released in 7 days (Fig. 5B). 

## Cytotoxicity Study

### *MTT assay*

**Figure 6:**
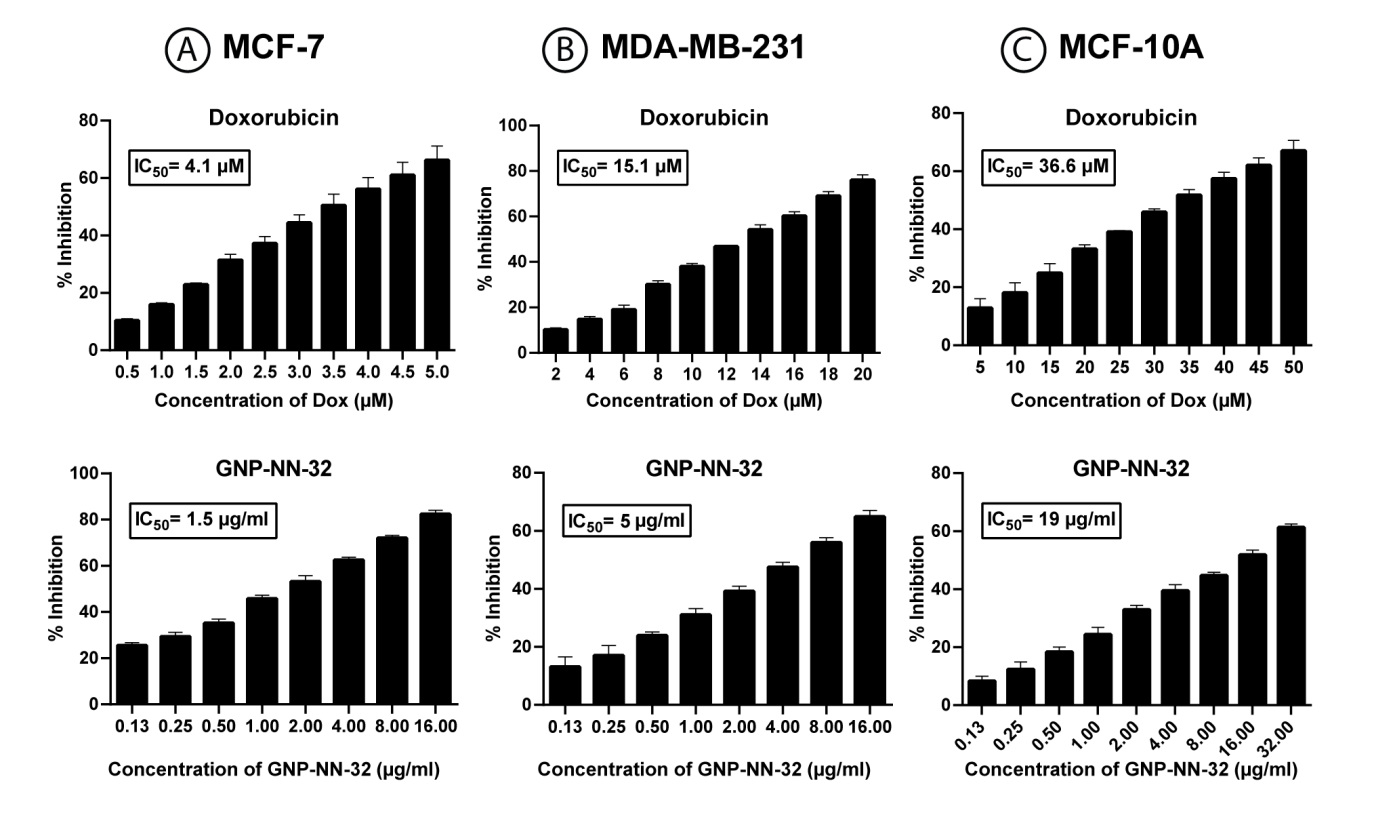
Inhibition of cells after 48 hours treatment of doxorubicin and GNP-NN-32. **(A)** MCF-7 cells, **(B)** MDA-MB-231 cells and **(C)** MCF-10A cells. Values are expressed as mean ± standard deviation (n = 3).

Cytotoxic activity of GNP-NN-32 toxin was assessed in relation to both estrogen receptor positive (ER+) (MCF-7 cells) and estrogen receptor negative cells (ER-) (MDA-MB-231 cells). The percent inhibition of growth of these cell lines after treatment with the GNP-NN-32 and doxorubicin after 48 hours was determined. The responses of MCF-7 and MDA-MB-231 cells to rising concentration of GNP-NN-32 and doxorubicin are shown in Figure 6A and 6B, respectively. The results showed that the inhibition of the aforementioned cell lines upon treatment with GNP-NN-32 and doxorubicin is concentration dependent.

The IC_50_ values after 48hours of treatment were found to be 4.1 µM and 15.1 µM for doxorubicin and 1.5 and 5.0 µg/mL for GNP-NN-32 with respect to MCF-7 and MDA-MB-231 cell lines, respectively. In case of normal breast epithelial cell lines (MCF-10A), the IC_50_ value of doxorubicin and GNP-NN-32 after 48 hours of treatment was found to be 39.6 µM and 19µg/mL, respectively which was about 10 times higher than that of the MCF-7 cells (Fig. 6C). Lower IC_50_ values shown by GNP-NN-32 for the cancer cells as compared to the normal breast cells suggest that the toxin could have great potential as an anti-cancer agent.

### *Anti-proliferation assay*

**Figure 7:**
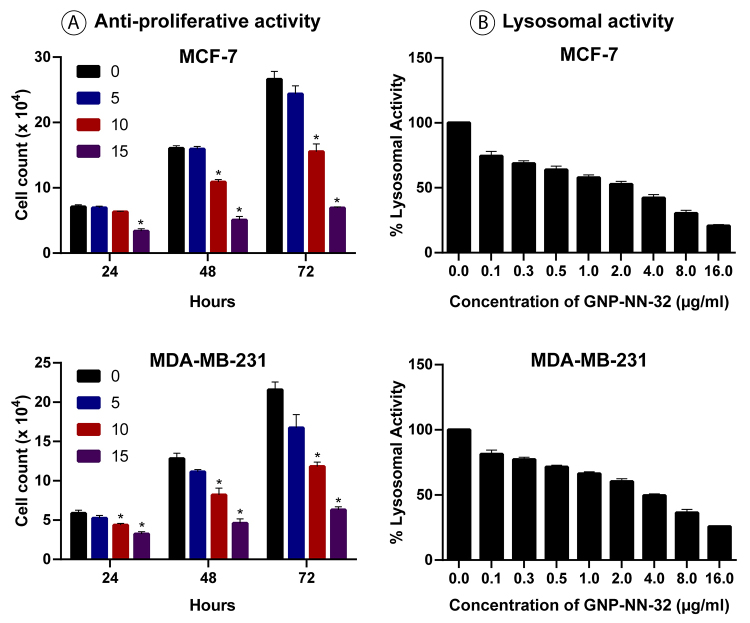
(A) Viability of MCF-7 and MDA-MB-231 cells treated with GNP-NN-32 after 24, 48 and 72 hours. (B) Lysosomal activity of MCF-7 and MDA-MB-231 cells treated with GNP-NN-32 toxin for 72 hours, determined by neutral red uptake assay. Values are expressed as mean ± standard deviation (n = 3). * p < 0.05 in comparison with the control group.

The anti-proliferative effects of the GNP-NN-32 toxin on MCF-7 and MDA-MB-231 cells were illustrated in Fig. 7A. The percent survival of the MCF-7 cells after 24, 48 and 72 hours of incubation with 10 µg/mL of GNP-NN-32 were 89, 59 and 48% respectively. However, MDA-MB-231 cells similarly treated with the GNP-NN-32 did not reveal much reduction in viability with values 74, 64 and 54% respectively as compared to that of MCF-7 cells. 

There was no significant change in cell count when cells were treated with lower concentration (5 µg/mL) of GNP-NN-32 at all the time intervals, but there was a significant reduction in MCF-7 and MDA-MB-231 cell count when treated with higher (10 µg/mL and 15 µg/mL) concentration in all the incubation periods. Therefore, GNP-NN-32 exhibited an anti-proliferative effect on both cancer cell lines used in the study.

## Neutral Red Uptake Assay

Both MCF-7 and MDA-MB-231 cell lines showed a significant decrease in lysosomal activity in a dose-dependent manner (Fig. 7B). The intensity of the neutral red staining is directly proportional to a few viable cells because, of its diffusion through cell membrane and accumulation in lysosomes.

## Apoptosis Study by Flow Cytometry

The typical representation of flow cytometric data showed as, lower left (LL) quadrant (Annexin V-/PI-) represents live cells, lower right quadrant (LR) (Annexin V+/PI-) is considered early apoptotic stage cells, upper right quadrant (UR) (Annexin V+/PI+) represents the late apoptotic stage cells and upper left quadrant (Annexin V-/PI+) represents cells in necrotic stage.

**Figure 8:**
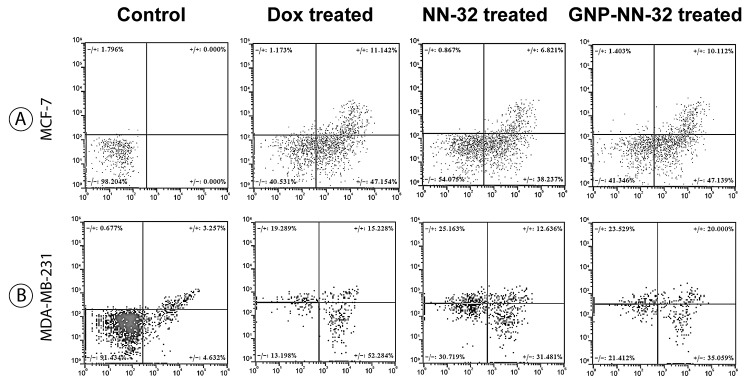
Flow cytometry analysis of apoptosis of human breast cancer cells stained with Annexin V-FITC PI, after treatment with doxorubicin, NN-32 and GNP-NN-32. **(A)** MCF-7 cells and **(B)** MDA-MB-231 cells.

Flow cytometric data analysis revealed that untreated control MCF-7 cells showed 98.2% live cells, 0% early apoptotic, 0% late apoptotic cells and 1.8% necrotic cells. Doxorubicin (standard drug) (IC_50_ dose) treatment for 48 hours showed 40.5% live cells, 47.1% early apoptotic, 11.1% late apoptotic and 1.2% necrotic cells. NN-32 (IC_50_ dose) treated showed 54.1% live cells, 38.2% early apoptotic, 6.8% late apoptotic and 0.86% necrotic cell. GNP-NN-32 (IC_50_ dose) treated showed 41.3% of cells live, 47.2% cells in early apoptotic, 10.1% cells in late apoptotic and 1.4% cells were in necrotic condition (Fig. 8A).

The control MDA-MB-231 untreated plate showed 91.4% live cells, 4.6% early apoptotic, 3.2% late apoptotic and 0.7% necrotic cells. Doxorubicin (standard drug) (IC_50_ dose) treated cells showed 13.2% live cells, 52.3% early apoptotic, 15.2% late apoptotic and 19.3% necrotic cells. After 48 hours of treatment of MDA-MB-231 cells with NN-32 (IC_50_ dose) the flow cytometric data analysis revealed 30.7% live cells, 31.5% early apoptotic, 12.6% late apoptotic and 25.2% necrotic cells. GNP-NN-32 (IC_50_ dose) treated cells showed 21.4% live cells, 35.1% cells in early apoptotic stage, 20% cells in late apoptotic and 23.5% cells were in necrotic condition (Fig. 8B).

These results revealed that NN-32 and GNP-NN-32 induced apoptosis in both MCF-7 and MDA-MB-231 cells after treatment. However, GNP-NN-32 showed higher apoptotic activity towards the aforementioned cell lines than that of the NN-32 alone which was similar to the standard drug doxorubicin.

## CFSE Cell Proliferation Study

**Figure 9:**
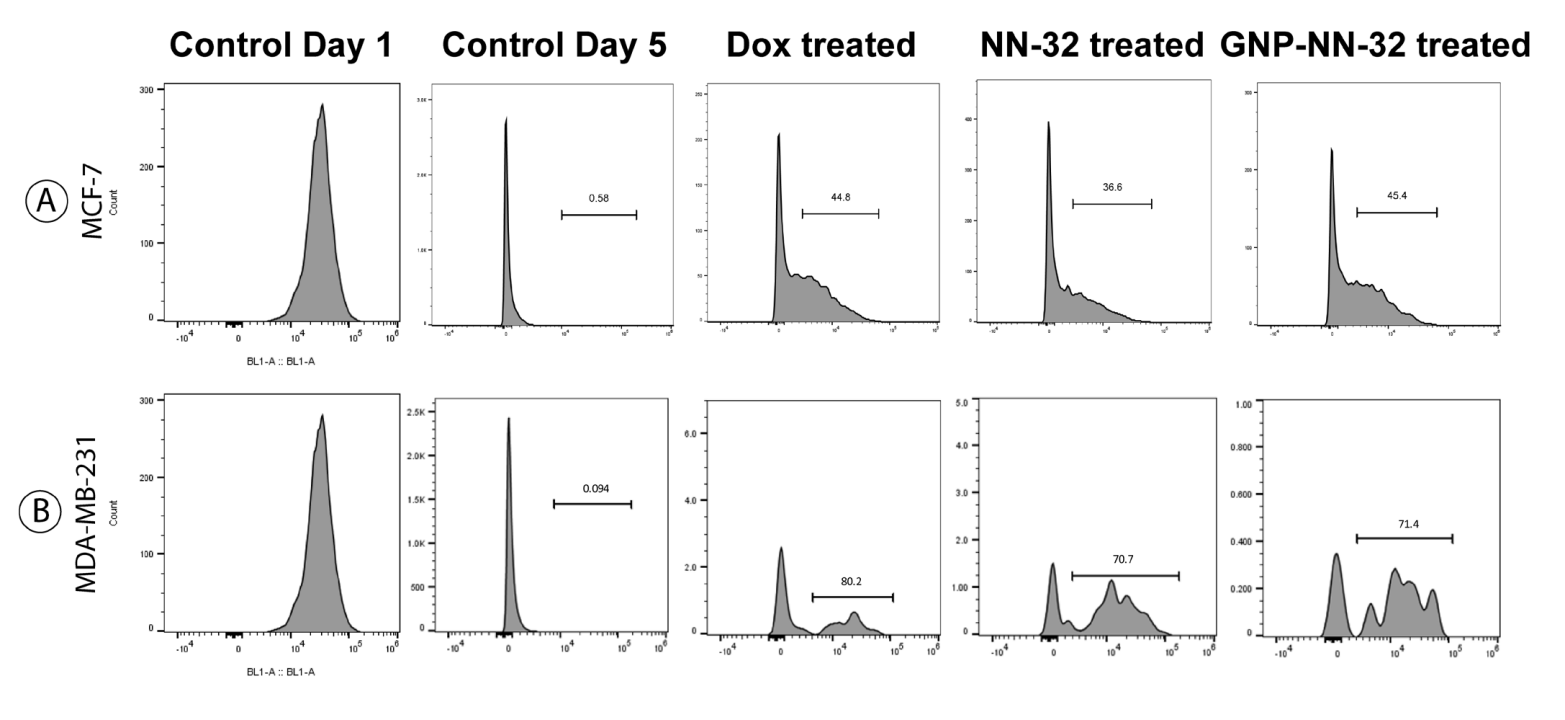
Cell proliferation study of human breast cancer cells stained with CFSE, after treatment with doxorubicin, NN-32 and GNP-NN-32. **(A)** MCF-7 cells and **(B)** MDA-MB-231 cells.

When cells were treated with NN-32, GNP-NN-32 and doxorubicin there was a halt in cell division so that we could see some peaks of CFSE in between after 5 days of incubation. This indicated that these agents inhibited cell cycle. At day 1, control graph showed all the cells with maximum CFSE intensity, but after 5 days intensity was reduced and reached to zero ultimately for both the cell lines. In case of MCF-7 cells (Fig. 9A), doxorubicin treated group showed 44.8% cells which were not divided normally and NN-32 and GNP-NN-32 treated group showed 36.6% and 45.4% cells respectively with halts in cell division respectively.

 MDA-MB-231 cells (Fig. 9B) also revealed similar results such as doxorubicin, NN-32 and GNP-NN-32 treated groups showed 80.2%, 70.7% and 71.4% cells with halts in cell division, respectively, and did not exhibit a normal pattern of cell division. The results of CFSE cell proliferation study revealed that NN-32 and GNP-NN-32 arrested cell division in both MCF-7 and MDA-MB-231 cell lines resulting in inhibition of proliferation of these cancer cells.

## Discussion

In this study, a cytotoxic snake venom protein toxin NN-32 (purified from *Naja naja* venom, found in chromatographic fraction no. 32, hence named NN-32) was tagged with gold nanoparticles with a polyethylene glycol (PEG) ligand protecting layer. PEG ligands were found to be very much effective in preventing nonspecific binding of peptides and enzymes to the gold nanoparticles [28]. PEG had been commonly used as a biomaterial owing to its low toxicity and immunogenicity [29]. PEG-coated GNP with functional carboxyl groups on their surface was formed and allowed for further controlled conjugation with NN-32 that avoided peptide-peptide interaction and aggregation within the nanoparticles [30]. The biomedical application of nanoparticles was restricted due to their spontaneous clearance from the *in vivo* system [31].

The present investigation confirmed the increased percentage of cytotoxic activity and apoptosis against two human breast cancer cell lines (MCF-7 and MDA-MB-231) after the conjugation of protein toxin NN-32 with gold nanoparticles. Gold nanoparticles have a special attraction to a sulfur atom [32]. In addition to cysteine residue of NN-32, PEG was utilized to ensure better stability of GNP NN-32. 

The anti-proliferative and the cytotoxic properties of GNP-NN-32 were confirmed by observations in cell growth inhibition studies and MTT. Imatib loaded microcapsules were found to be superior than native imatib in respect of inhibition of growth of K562 cells in a study conducted by Palamàet al. [33]. A similar approach was also employed by Luo et al. [34] and Yang et al. [35] who used n-succinyl chitosan nanoparticles and lipid nanoparticles on K562 cells for better antitumor effect.

The compounds act as anti-tumor agents at multiple steps in the cell cycle and their effects may be either cytostatic or cytotoxic, depending on the cell cycle status of the target cells [36]. In this context, this study indicated that GNP-NN 32 was capable of inducing apoptosis at a concentration of 1.5 μg/mL in MCF-7 cell line and 5 μg/mL in MDA-MB-231 cell line. Our results are in agreement with other studies that discovered that disintegrin from *Crotalus durissus collilineatus* venom induced cytotoxicity [37] whereas BthTX-I from *Bothrops jararacussu* induced apoptosis [38] and also showed antitumor and antimetastatic effects [39] on human breast cancer cell lines.

Apoptosis is a continuous and highly regulated cellular process resulting in the destruction of undesirable cells during developmental stages or homeostasis in multicellular organisms [40]. The process of apoptosis is characterized by significant changes such as nuclear fragmentation, chromatin condensation and membrane blebbing. Snake venom cytotoxins were reported to bind zwitterionic or acidic phospholipid of a membrane leading to aggregation or fusion of phospholipid vesicles [41]. It was also reported that cytotoxins affected the function of membrane proteins such as Na+/K+-ATPase, protein kinase C, and integrins [42, 43, 44].

Phosphatidylserine (PS) is the key substance for detection of apoptosis by flow cytometry. In a normal healthy cell, PS presents in inner leaflet of the membrane, whereas when cell undergoes apoptosis, itis externalized onto the outer leaflet of the membrane whereby it can now bind to Annexin V in presence of calcium. This binding is detected by flow cytometry as Annexin V positive cells. Flow cytometry data showed the dot plot, supporting the fact that treatment with GNP-NN-32 induced apoptosis in MCF-7 and MDA-MB-231 cancer cell lines. Cells undergoing apoptosis usually exhibit fragmentation of the cell into membrane-bound apoptotic bodies, nuclear and cytoplasmic condensation and endolytic cleavage of the DNA into small oligonucleosomal fragments [45].

The altered expression of two distinct genes responsible for regulating the mitochondrial pathway of apoptosis are antiapoptotic Bcl-2 and proapoptotic [45]. NKCT1 toxin from *Naja kaouthia* venom induced apoptosis in U937 and K562 leukemic cell lines through activation of Bax: Bcl2 [9]. 

The actual mechanism of action of these snake venom toxins is by mitochondria dependent cell death pathway, wherein it upregulates expression of pro-apoptotic Bax and down-regulates expression of anti-apoptotic Bcl2, which internally leads to the increase in permeability and release of cytochrome c from mitochondrial membrane [46]. The release of cytochrome c triggered the caspase activation via oligomerization of APAF1 protein. APAF1 converts pro-caspase 9 to active caspase 9. Caspase 9 further activated caspase 3 from pro-caspase 3. Caspase 3 is an effector downstream molecule that triggers the cascade of intracellular events leading to programmed cell death.

## Conclusion

The purified toxin NN-32 and nanogold conjugated toxin GNP-NN-32 exhibited significant cytotoxic potential against breast cancer cell lines (MCF-7 and MDA-MB-231) in positive correlation with dose and duration of exposure. After conjugation with gold nanoparticles, the increased efficacy of GNP-NN-32 was inferred from the lower IC_50_ values. NN-32 and GNP-NN-32 induced apoptosis and arrested cell cycle of both MCF-7 and MDA-MB-231 cell lines. However, further detailed studies are required in this area. Snake venoms are a cocktail of several toxic components that have great potential to be used along with nanotechnology as therapeutic agents against cancer and other diseases. Coordinated efforts from the scientists, clinicians, research and development industry are necessary for the development of potential drugs in the near future.

### Abbreviations

CFSC: 5(6)-carboxyfluorescein diacetate N-succinimidyl ester; DLS: dynamic light scattering; DMEM: Dulbecco’s modified eagle medium; DMSO: Dimethylsulphoxide; EAC: Ehrlich ascites carcinoma; ER-: estrogen receptor negative; ER+: estrogen receptor positive; FBS: fetal bovine serum; IC_50_: half maximal inhibitory concentration; LDH: lactate dehydrogenase; MTT: 3-(4,5-dimethylthiazol-2-Yl)-2,5-diphenyltetrazolium bromide; NP: nanoparticles; NR: neutral red; PEG: polyethylene glycol; PI: propidium iodide; PS: phosphatidylserine; RBC: red blood cells; TEM: transmission electron microscopy; β-NADH: β-nicotinamide adenine dinucleotide reduced dipotassium salt.
